# The strengths, weaknesses, opportunities, and threats of generative artificial intelligence: a qualitative study of undergraduate nursing students

**DOI:** 10.3389/fpubh.2025.1672140

**Published:** 2025-09-04

**Authors:** You Yuan, Jing Fu, Lanlan Leng, Zhuosi Wen, Xiaoman Wei, Die Han, Xinyang Hu, Yu Liang, Qian Luo, Xia Zhang, Rujun Hu

**Affiliations:** ^1^Department of Critical Care Medicine, Affiliated Hospital of Zunyi Medical University, Zunyi, Guizhou, China; ^2^Department of Nursing, Affiliated Hospital of Zunyi Medical University, Zunyi, Guizhou, China; ^3^School of Nursing, Zunyi Medical University, Zunyi, Guizhou, China; ^4^Department of Critical Care Medicine, Zhejiang Provincial People’s Hospital Bijie Hospital, Bijie, Guizhou, China; ^5^Department of Nursing, Zunyi Medical and Pharmaceutical College, Zunyi, Guizhou Province, China

**Keywords:** generative artificial intelligence, undergraduate nursing students, SWOT analysis, education, qualitative study

## Abstract

**Background:**

While Generative Artificial Intelligence (Gen AI) is increasingly applied in nursing education, research on undergraduates’ perceptions, experiences, and impacts remains limited.

**Objective:**

This study aims to explore undergraduate nursing students’ perceptions of the strengths, weaknesses, opportunities, and threats (SWOT) associated with Gen AI through qualitative research methods.

**Methods:**

Using the SWOT analysis framework as the theoretical basis, data were collected through semi-structured interviews with nursing undergraduates via convenience sampling from May to July 2025 until saturation, and analyzed using Colaizzi’s phenomenological method for thematic extraction.

**Results:**

A total of 36 nursing undergraduates were interviewed, from whom four main themes and 16 sub-themes were identified. These were categorized into internal and external factors. Internal positive factors (Strengths) included personalized learning assistance, skill training and curriculum support, efficiency and cognitive expansion, and data processing and learning capability. Internal negative factors (Weaknesses) involved ethical and legal risks, the generation of low-quality or inaccurate outputs, technical barriers, and cognitive and learning risks. External opportunities comprised policy and resource support, technological advancement and evolution, interdisciplinary integration and collaboration, and emerging career opportunities. External threats included technological adaptation and cost risks, digital divide and equity gap, job displacement risk, and educational integrity risk.

**Conclusion:**

Undergraduate nursing students regard generative AI as a double-edged sword—its strengths in boosting learning efficiency, broadening knowledge access and simulating clinical decisions are offset by ethical, technological and equity challenges. Nursing education must therefore strengthen technical guidance, ethics training and resource optimization to maximize its strengths and opportunities while minimizing its weaknesses and threats.

## 1 Introduction

In recent years, Generative Artificial Intelligence (Gen AI) has rapidly developed into a major branch of artificial intelligence (1, 2). Powered by large-scale language models and related technologies, Gen AI is capable of automatically generating text, images, videos, audio, and multi-modal content. It has been widely applied across various sectors, including education, healthcare, and finance (3–5). With the increasing popularity of representative models such as ChatGPT, Gemini, and Claude, Gen AI has gradually entered higher education settings, driving profound changes in educational models (6). In the field of nursing education, Gen AI is expected to serve as an auxiliary tool for both teaching and practice, enhancing students’ learning efficiency, expanding access to knowledge, and, to some extent, simulating clinical decision-making processes—thus supporting the modernization of nursing talent cultivation (7–9).

However, the rapid integration of this technology has also introduced new challenges. Existing studies have pointed out that AI in education may lead to student over-reliance, reduced ability to critically assess learning content, and even a decline in critical thinking skills (10–13). Moreover, the accuracy, professional consistency, and ethical implications of AI-generated content—particularly in medical and health-related disciplines—are issues of significant concern (14–18). For undergraduate nursing students, who are both direct users of this emerging technology and future front-line clinical professionals, it is crucial to understand their perceptions of the strengths, weaknesses, opportunities, and potential threats associated with Gen AI. Such understanding is essential for promoting the effective integration of AI into nursing education and for formulating targeted teaching strategies (19).

Although some quantitative studies have explored nursing students’ acceptance and usage of AI, in-depth insights into their subjective experiences, specific perceptions, and the educational impact of Gen AI remain limited (20–24). Particularly in nursing education practice, there is a lack of a systematic theoretical framework to evaluate both the benefits and risks introduced by Gen AI. To address this research gap, the present study adopts a qualitative research approach, using the SWOT analysis framework (Strengths, Weaknesses, Opportunities, Threats) as its theoretical foundation. Through in-depth interviews with undergraduate nursing students, this study systematically explores their perceptions and experiences of using generative AI.

## 2 Methods and procedures

### 2.1 Study design

To gain an in-depth understanding of undergraduate nursing students’ perceptions and experiences with Gen AI, this study conducted a systematic qualitative investigation grounded in preliminary quantitative findings. In the initial phase, the research team developed and refined a structured questionnaire covering five dimensions: demographic information, AI tool usage, knowledge level, attitudes, and perceived challenges. Following three rounds of expert review by five nursing education specialists and two AI experts, the finalized questionnaire was administered to 2,340 undergraduate nursing students at Zunyi Medical University, representing various regions. A total of 567 valid responses were received. Although the quantitative data were analyzed separately and are not reported in this article, they provided essential background information. Participants for the qualitative phase were purposively recruited from those who had indicated willingness to engage in follow-up interviews, thereby informing the design and direction of the subsequent qualitative inquiry.

Building upon the quantitative results, the second phase adopted a qualitative phenomenological approach to explore students’ subjective experiences and perceptions related to Gen AI use, focusing on four key dimensions: Strengths, Weaknesses, Opportunities, and Threats (SWOT). Semi-structured interviews were conducted between May and July 2025 at Zunyi Medical University. Participants were selected through convenience sampling from those who had indicated their willingness to participate in interviews in the initial survey. Recruitment continued until data saturation was achieved.

This study was approved by the Ethics Committee of the Affiliated Hospital of Zunyi Medical University (Approval No. KLL-2025-069) and strictly adhered to the ethical principles outlined in the Declaration of Helsinki. All participants provided written informed consent. The study design and reporting followed the Consolidated Criteria for Reporting Qualitative Research (COREQ) guidelines.

### 2.2 Theoretical framework

The SWOT model, developed by Albert Humphrey, is a structured strategic analysis tool widely applied in management, policy, and educational research (24–27). By identifying internal factors (Strengths and Weaknesses) and external factors (Opportunities and Threats), the model provides a theoretical foundation for strategic planning and resource allocation (25).

In this study, the SWOT model was adopted as the analytical framework to examine the application of Gen AI in nursing education systematically. Semi-structured interviews were conducted to capture the authentic perceptions and experiences of undergraduate nursing students, and the qualitative data were coded and categorized to identify the strengths, weaknesses, opportunities, and threats associated with their use of Gen AI.

Internal factors reflected individual-level experiences and evaluations. Strengths included personalized learning, enhanced autonomy, improved learning efficiency, and cognitive training; weaknesses encompassed technological barriers, difficulties in understanding, reduced initiative, increased dependency, and ethical concerns. External factors refer to environmental influences. Opportunities involved policy support, technological advancement, improved digital literacy, and intelligent resource development; threats included regulatory lag, ethical controversies, urban–rural disparities, and issues related to educational equity.

Based on the SWOT findings, a 2 × 2 strategic matrix was constructed to propose four strategic pathways: SO strategies (leveraging strengths to seize opportunities), ST strategies (leveraging strengths to mitigate threats), WO strategies (overcoming weaknesses to exploit opportunities), and WT strategies (overcoming weaknesses to avoid threats).

**Figure 1:**
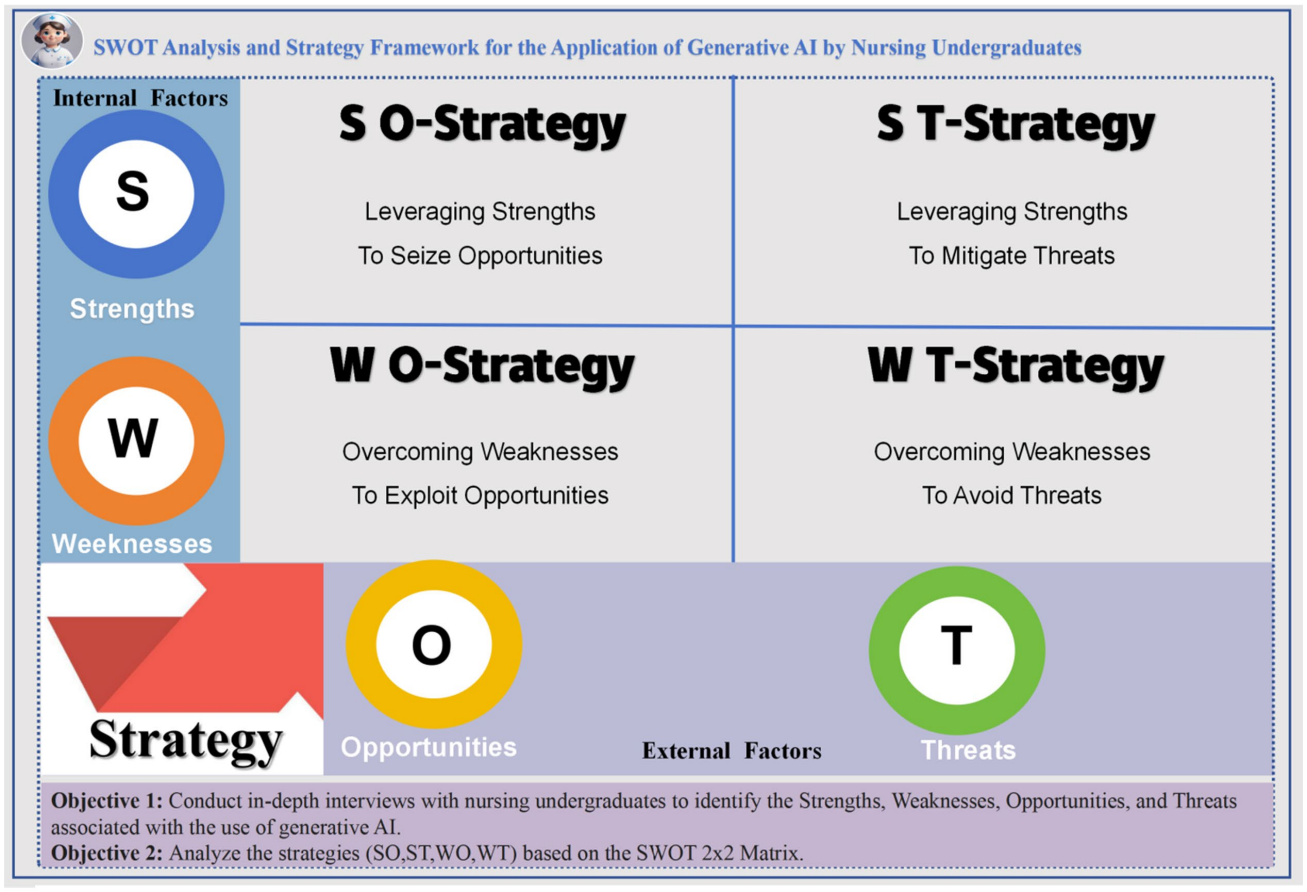
SWOT analysis and strategy framework for the application of Gen AI by nursing undergraduates.

The integration of this theoretical framework not only enabled a systematic identification of the four core dimensions facing Gen AI in nursing education but also enhanced the explanatory power and practical relevance of the study. It provides a theoretical foundation and practical road map for the future integration of AI technologies into educational settings (Figure 1).

### 2.3 Interview guide and inclusion/exclusion criteria

Before the formal interviews, the research team conducted an extensive review of both domestic and international literature related to Gen AI and nursing education. Based on the findings from the preliminary quantitative survey and guided by qualitative research design principles, a draft interview guide was developed. After multiple rounds of expert consultation and revision, a finalized semi-structured interview guide was established, structured around the four core dimensions of the SWOT framework (Table 1). SWOT analysis was employed solely as a framework for data organization and theme development by the research team. The interviewees did not receive any training related to SWOT; instead, their participation focused only on expressing their views on the strengths, weaknesses, opportunities, and threats of applying AI in nursing education.

**Table 1:** Interview outline centered on the four core dimensions of SWOT.

Interview questions	SWOT dimension	Purpose of inquiry
What is your overall impression of Gen AI?	General (S/W/O/T)	To gain initial understanding of students’ perceptions and attitudes as general context.
What are the main reasons you use Gen AI?	Strengths	To explore motivations for use and the positive roles AI plays in the learning process.
What do you find most helpful about Gen AI in your studies?	Strengths	To focus on the key value of AI in improving learning efficiency, quality, or abilities.
What specific difficulties have you encountered when using Gen AI?	Weaknesses	To identify operational, comprehension, or accessibility challenges experienced.
What features or functions of AI tools do you find most difficult to use or understand?	Weaknesses	To investigate technical barriers or design shortcomings in the user experience.
How do you think Gen AI can further support your learning in the future?	Opportunities	To gather students’ expectations and suggestions regarding future AI applications.
What kind of support or resources would you most like to have to better use Gen AI?	Opportunities	To capture practical needs related to educational resources, platform features, or training.
Are you concerned about any negative impacts of using Gen AI?	Threats	To explore concerns about dependence, academic misconduct, or potential misuse.
If misused, what specific negative effects do you think Gen AI might have on learning?	Threats	To clarify potential consequences such as passive thinking, misinformation, or learning decline.
I have no further questions. Is there anything you would like to add?	General (S/W/O/T)	To encourage students to share additional valuable perspectives not covered above.

Interviews were conducted face-to-face in quiet and private settings such as libraries or classrooms. Before each interview, the researcher clearly explained the study’s objectives, procedures, and confidentiality protocols to the participants. It was emphasized that all interview content would be used solely for academic research purposes. Written informed consent was obtained before audio recording.

During the interviews, the researcher employed techniques such as clarification, probing, repetition, and summarization to encourage participants to articulate their perspectives and usage experiences in depth. Each interview lasted approximately 10 to 60 min. Before concluding, the interviewer asked: “I have no further questions. Is there anything you would like to add?” to ensure participants had ample opportunity to share their views.

This study adopted a maximum variation sampling strategy to ensure diversity across variables such as academic year, gender, and urban–rural background. Inclusion criteria required participants to be current undergraduate nursing students who had completed the prior questionnaire and voluntarily agreed to participate in the interview. Exclusion criteria included students who were currently on leave, enlisted in military service, or otherwise not enrolled, as well as those unwilling to engage in qualitative interviews. Data collection continued until thematic saturation was reached, meaning no new perspectives or themes emerged.

### 2.4 Data analysis and quality control

This study employed Colaizzi’s seven-step phenomenological analysis method to systematically analyze semi-structured interview data from undergraduate nursing students, aiming to gain an in-depth understanding of their lived experiences and perceptions regarding the use of Gen AI.

All interviews were conducted in Chinese. During recording, we used the iFlytek AI voice recorder SR502, which has speech-to-text capability and can effectively recognize dialects. Within 24 h after each interview, two research assistants trained in qualitative methods checked the verbatim transcripts, and any discrepancies were resolved by a third researcher. Subsequently, two bilingual team members with medical backgrounds translated the meaningful Chinese statements into English and employed a back-translation approach to ensure semantic and cultural accuracy. The resulting bilingual files were securely encrypted to maintain data integrity and confidentiality.

The data analysis followed these steps: (1) researchers repeatedly read through the transcripts to gain a holistic understanding; (2) significant statements directly related to the research topic were identified and extracted; (3) these statements were condensed to formulate meaningful units; (4) the units were open-coded and initially categorized; (5) the emerging themes were then organized according to the four dimensions of the SWOT framework—Strengths, Weaknesses, Opportunities, and Threats; (6) contents were further synthesized to construct a comprehensive structure that reflected the participants’ collective perspectives; (7) the results were returned to selected participants for member checking to validate their accuracy and representativeness.

To ensure research rigor and analytical reliability, all interviewers received standardized training in qualitative interviewing techniques. Initial coding and theme development were independently conducted by four researchers, followed by multiple rounds of team discussions to achieve consensus. An audit trail and analytic memos were maintained throughout the process to ensure transparency and traceability.

To enhance credibility, original participant quotes were preserved during analysis, and conclusions were verified through member checking. Maximum variation sampling was applied, covering diverse academic years, genders, and urban–rural backgrounds to increase the study’s breadth and transferability. Ultimately, the research team synthesized the thematic findings into a SWOT-based 2 × 2 strategic matrix, offering a structured presentation of integration pathways and responsive strategies for Gen AI in nursing education. This approach strengthens both the theoretical depth and practical relevance of the study.

## 3 Results

A total of 36 undergraduate nursing students were included in this qualitative study. The average age of participants was 20.28 ± 1.41 years. The cumulative interview duration reached 1,421 min, with an average length of 39.47 ± 10.53 min per interview. Female students accounted for 72.22%, and the distribution across academic years was as follows: Freshman (25.00%), Sophomore (30.56%), Junior (22.22%), and Senior (22.22%). In terms of background, 36.11% were from urban areas, and 69.44% reported previous experience participating in research projects.

Regarding the use of generative AI tools, 80.56% of students had experience with three or more platforms, with a strong preference for domestic tools. The most commonly used tools included Deep Seek (91.67%), Dou bao and Quark (each at 63.89%), Uni-Search (36.11%), Kimi (25.00%), and ChatGPT (11.11%). In terms of frequency, 38.89% of students used AI tools multiple times per day, and 33.33% reported using them 3–5 times per week. As for duration, 36.11% used AI tools for less than 15 min per session, 44.44% for 15–30 min, and 19.44% for more than 30 min.

**Figure 2:**
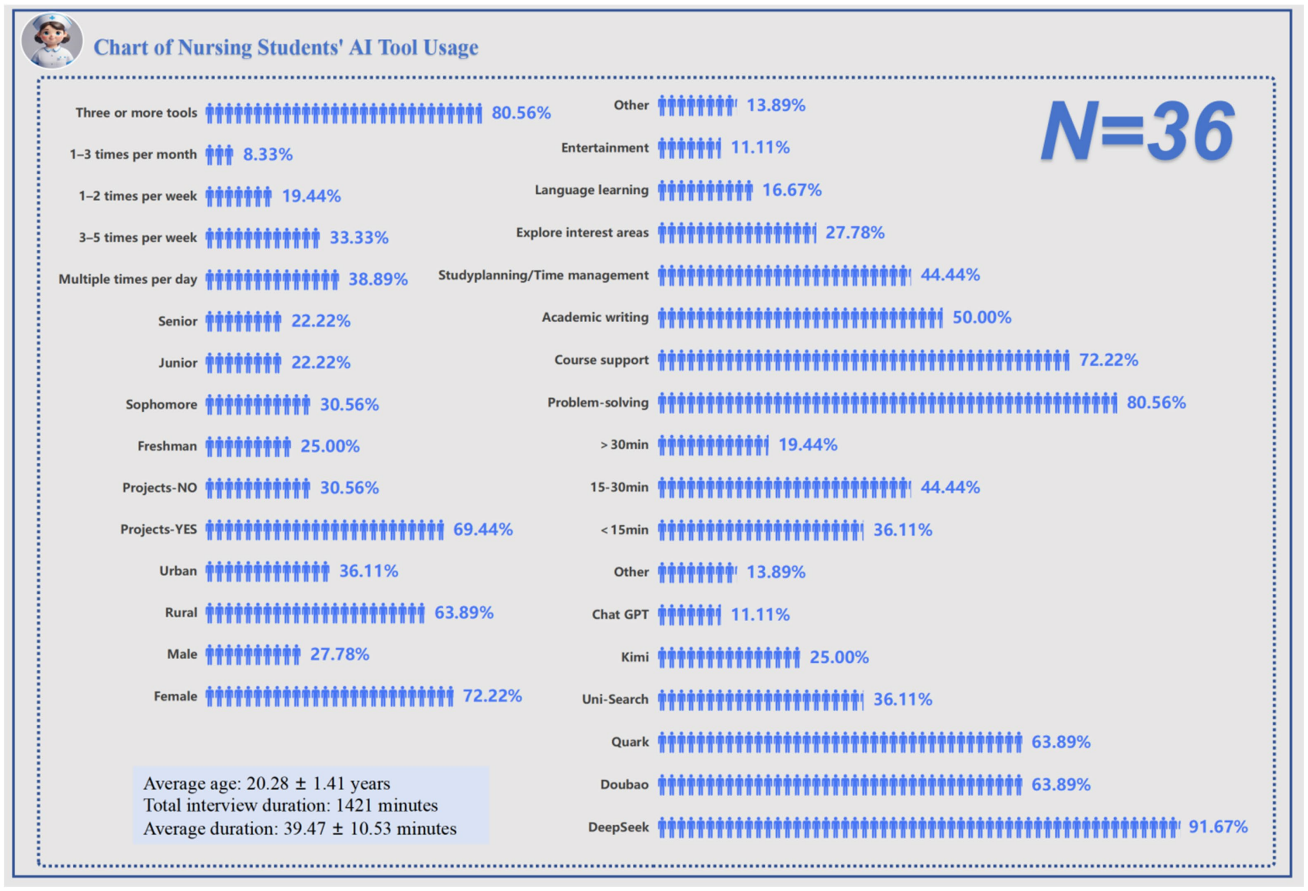
Chart of nursing students’ AI tool usage (*N* = 36).

Students primarily used AI tools for academic purposes, including problem solving (80.56%), course support (72.22%), and academic writing (50.00%). Other uses included study planning (44.44%), interest exploration (27.78%), language learning (16.67%), and entertainment (11.11%). Overall, undergraduate nursing students demonstrated a strong tendency to use generative AI tools extensively in academic contexts, with a clear preference for domestic platforms (Table 2; Figure 2).

**Table 2:** Basic information of interviewed students (*N* = 36).

No.	Grade	Gender	Age	Residence	Used AI tools	Usage frequency	Usage duration	Usage purpose	Projects	Interview time
S1	Freshman	Female	19	Rural	①②③	Multiple times per day	<15 min	①②③	YES	46
S2	Junior	Female	20	Urban	①②⑤	3–5 times per week	>30 min	①②④	NO	37
S3	Sophomore	Female	20	Rural	①③④	3–5 times per week	15–30 min	①②④	NO	34
S4	Senior	Female	21	Urban	①③⑤	Multiple times per day	15–30 min	①②③⑦	YES	39
S5	Junior	Female	22	Urban	①②③⑥⑦	1–2 times per week	>30 min	①②③⑤	YES	55
S6	Sophomore	Male	20	Rural	①②④	Multiple times per day	<15 min	①②③⑤	YES	44
S7	Freshman	Female	18	Urban	①②⑤	3–5 times per week	15–30 min	①⑥	NO	31
S8	Freshman	Female	19	Rural	①③	1–2 times per week	>30 min	①⑧	NO	21
S9	Freshman	Female	18	Rural	①②⑤	Multiple times per day	15–30 min	②④⑥	NO	47
S10	Sophomore	Male	20	Rural	①②③	3–5 times per week	15–30 min	②③⑥	YES	38
S11	Senior	Male	23	Urban	①③④	3–5 times per week	<15 min	①②④⑤	YES	43
S12	Sophomore	Female	19	Urban	①②④	3–5 times per week	<15 min	①②③④	YES	47
S13	Freshman	Female	19	Rural	①③④	Multiple times per day	15–30 min	①②⑦	YES	45
S14	Junior	Female	21	Rural	①②	1–2 times per week	15–30 min	①③	YES	27
S15	Junior	Female	22	Rural	①②③	Multiple times per day	<15 min	①②③⑤⑥	YES	45
S16	Sophomore	Female	20	Rural	①②③	Multiple times per day	15–30 min	①②③④	YES	53
S17	Freshman	Female	18	Urban	①②④	3–5 times per week	15–30 min	②④	NO	30
S18	Senior	Male	22	Rural	②④	1–2 times per week	>30 min	①②	YES	29
S19	Freshman	Female	18	Rural	①③	1–2 times per week	<15 min	①③⑧	YES	31
S20	Junior	Male	21	Urban	①②③④⑥	3–5 times per week	15–30 min	①②③⑤	YES	51
S21	Senior	Female	22	Rural	①②④	Multiple times per day	<15 min	①②③⑤	YES	49
S22	Freshman	Female	19	Rural	①③④	3–5 times per week	<15 min	①②④	NO	32
S23	Junior	Female	21	Urban	①②③	Multiple times per day	15–30 min	①②③	YES	43
S24	Sophomore	Female	20	Rural	⑦	1–3 times per month	15–30 min	②	NO	14
S25	Sophomore	Male	21	Rural	①③	1–2 times per week	<15 min	②③	YES	26
S26	Junior	Female	21	Rural	①③⑤	Multiple times per day	15–30 min	①③④⑦	YES	44
S27	Sophomore	Female	20	Rural	⑤⑦	1–3 times per month	>30 min	⑤	YES	16
S28	Sophomore	Female	19	Urban	①③⑤	Multiple times per day	<15 min	①②⑦	YES	41
S29	Senior	Male	22	Urban	①②③④⑤⑥⑦	1–2 times per week	<15 min	①③⑤⑧	YES	52
S30	Senior	Female	22	Rural	①②③	Multiple times per day	15–30 min	①②④	YES	37
S31	Sophomore	Male	20	Rural	①②③	3–5 times per week	15–30 min	①②④	YES	42
S32	Sophomore	Female	20	Rural	①②④	Multiple times per day	>30 min	①②④⑥	NO	49
S33	Freshman	Female	19	Urban	①③④	3–5 times per week	<15 min	①④⑥	NO	39
S34	Senior	Female	23	Rural	①②③	Multiple times per day	15–30 min	①②③④⑤⑧	YES	54
S35	Junior	Male	20	Urban	①②⑤	3–5 times per week	<15 min	①④	NO	38
S36	Senior	Male	21	Rural	①②③⑥⑦	1–3 times per month	>30 min	③⑤⑧	YES	52

Used AI tools: ① Deep Seek; ② Doubao; ③ Quark; ④ Uni Search; ⑤ Kimi; ⑥ ChatGPT; and ⑦ Other. Usage purpose: ① Problem-solving; ② Course support; ③ Academic writing; ④ Study planning/time management; ⑤ Explore Interest Areas; ⑥ Language learning; ⑦Entertainment; and ⑧ Other.

**Figure 3:**
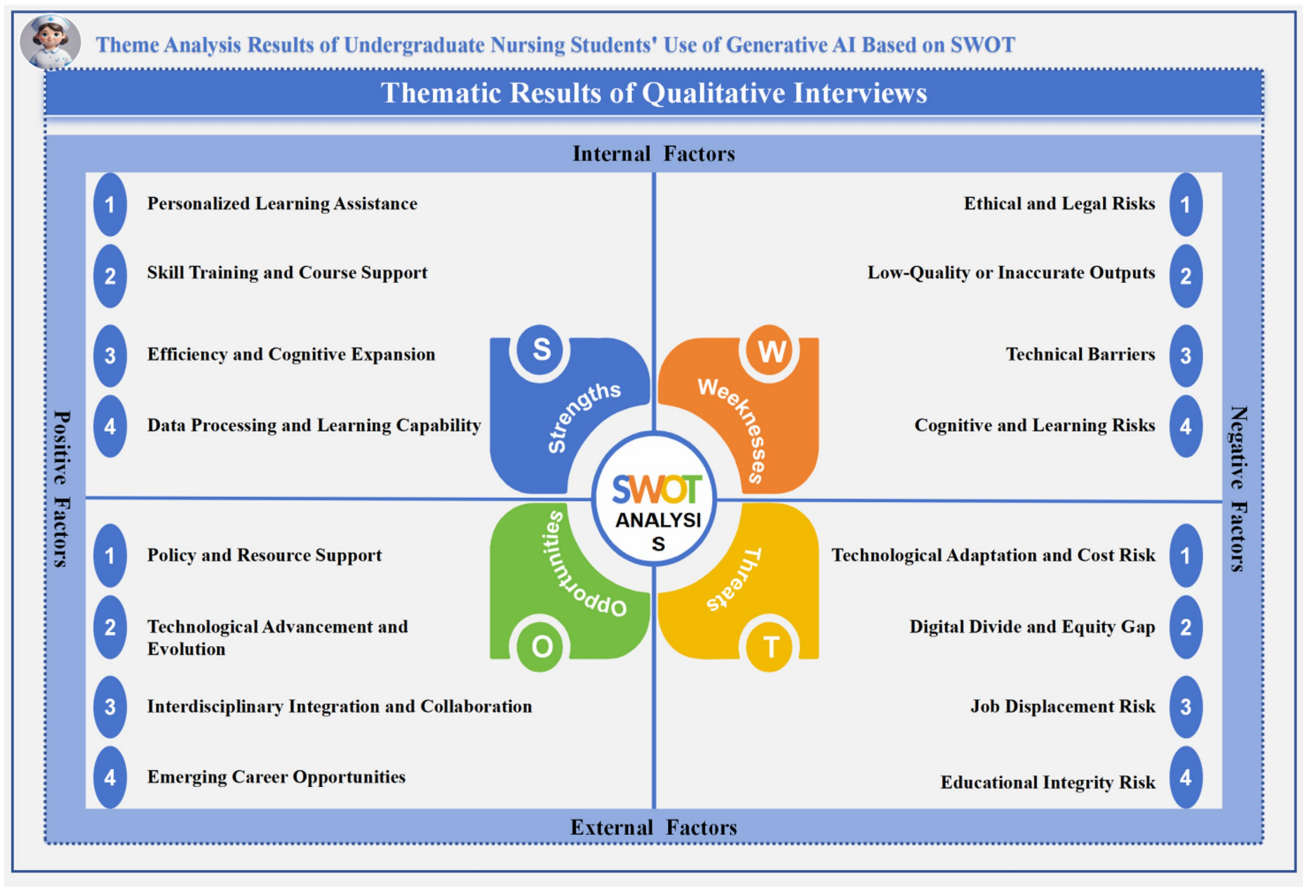
Theme analysis results of undergraduate nursing students’ use of Gen AI Based on SWOT.

Through in-depth interviews and systematic coding based on the SWOT framework, this study identified four main themes and 16 corresponding sub-themes (Figure 3). The findings comprehensively reveal the strengths, weaknesses, opportunities, and threats experienced by nursing students when engaging with generative AI, shedding light on the key challenges and potential value of integrating AI into nursing education. These results provide valuable insights for future research and pedagogical innovation in this domain.

### 3.1 Theme 1: strengths

In the in-depth interviews with undergraduate nursing students, the vast majority expressed strong recognition of the supportive role of Gen AI in their learning. They identified significant advantages in personalized learning, skill training, learning efficiency, and knowledge expansion.

#### 3.1.1 Personalized learning assistance

Gen AI was widely regarded as a nonjudgmental and interactive learning companion. Many participants noted that it provided a low-anxiety and highly inclusive environment, allowing them to ask questions freely and practice repeatedly—something they found difficult in traditional classroom settings due to shyness or fear of judgment:

*“I can ask AI the questions I’m too shy to ask my teachers—it will not judge me or get annoyed if the question is too simple.” (S1).*

*“When answering my questions, AI also refers to the ones I asked before. I feel it can be trained to suit my learning style.” (S16).*

Several students reported that Gen AI significantly enhanced their initiative and communication skills in learning:

*“I can ask questions anytime based on my needs, and it answers step by step patiently.” (S22).*

*“Compared to just listening to lectures, I enjoy interacting with AI more—it’s more engaging and helps me focus.” (S6).*

*“Sometimes I do not know how to phrase things, and it gives me sentence suggestions that help clarify my thoughts.” (S9).*

In academic writing, Gen AI was seen as a targeted and efficient tool:

*“When writing papers, AI helps me organize my ideas and structure, which improves my writing efficiency.” (S21).*

*“It helps me refine the logic of my essays, making the structure much clearer.” (S4).*

Additionally, some students believed AI expanded their learning beyond the textbook and broadened their horizons:

*“The textbook is limited, but AI lets me explore more extracurricular knowledge—things you do not get in class.” (S23).*

*“It explains problems from multiple angles, even touching on things that teachers might not mention.” (S9).*

#### 3.1.2 Skill training and course support

Some students described Gen AI as an “always-available tutor” that breaks time and space constraints by providing instant support throughout the learning process:

*“Gen AI breaks time limitations. It’s like a 24/7 teacher that gives free help whenever I need it.” (S4).*

In clinical skills learning, students acknowledged its potential in simulation and communication training:

*“It can simulate nurse–patient dialogues, which helps me practice communication skills and clinical response.” (S23).*

Gen AI was also frequently used for course comprehension and exam review:

*“Gen AI helps with understanding course content, especially complex concepts.” (S6).*

*“I often use it to review for exams—the information it provides is very useful.” (S10).*

*“It’s like a study buddy—it makes revision less boring.” (S7).*

Some noted that its conversational nature encouraged critical thinking and divergent thought:

*“Through dialogue—like follow-up questions on a case, it stimulates deeper thinking and helps train my divergent thinking.” (S26).*

#### 3.1.3 Efficiency and cognitive expansion

Many students emphasized that Gen AI greatly reduced the time spent on searching and organizing information, thereby improving overall learning efficiency:

*“I like to list out all the questions I do not understand, use the photo feature to upload them, and AI gives me quick answers—it saves a lot of time.” (S12).*

*“When study tasks pile up, it helps save so much time finding information.” (S15).*

At the same time, its strong information integration capabilities helped students view problems from multiple perspectives:

*“When I use it to look up a problem, it not only solves it but also pushes related concepts—very systematic.” (S17).*

*“Its answers are often more comprehensive than mine and inspire me to think in new ways.” (S13).*

In some classrooms, instructors encouraged a “human–AI collaborative” thinking model:

*“Sometimes the teacher asks us to answer first, then use AI to expand—it helps with reflection and idea development.” (S22).*

#### 3.1.4 Data processing and learning capability

Participants generally believed that Gen AI has powerful data processing and knowledge integration abilities, responding quickly to inputs and providing high-quality feedback:

*“It can identify key words from my question, explain complex concepts from different angles, and help me master the material faster.” (S9).*

*“Its database is extensive—it covers research articles, images, and videos, and the search speed is amazing.” (S28).*

In terms of multi-modal content generation, AI also showed strong adaptability:

*“Once I uploaded an image and gave a video reference, it generated what I needed, which felt very intelligent.” (S7).*

*“As long as I describe it enough, it understands what I want and recommends related videos.” (S17).*

Some students experimented with combining Gen AI and other tools to handle complex tasks with hybrid intelligence:

*“I saw someone use Gen AI with Deep Seek and PubMed to generate a review outline—it showed strong learning and integration abilities.” (S3).*

### 3.2 Theme 2: weaknesses

Although most nursing students acknowledged the positive role of Gen AI in their learning processes, many participants also expressed concerns and difficulties during actual use. These concerns mainly centered around ethical and legal risks, the quality of generated information, technical barriers, and potential negative effects on cognitive development and learning habits.

#### 3.2.1 Ethical and legal risks

Some students voiced concerns about the unclear delineation of legal responsibility and ethical boundaries when AI is involved in healthcare or learning-related decisions:

*“If a medical incident occurs during human-AI collaboration, who is ultimately responsible? Also, how do we balance AI’s decision-making with the authority of doctors?” (S18).*

The limitations of AI in simulating human empathy and emotional interactions were also frequently mentioned:

*“Although Gen AI improves efficiency, it cannot replace the emotional communication between people.” (S14).*

*“It just mechanically reminds patients to take their medication—it cannot patiently listen to their stories or provide emotional comfort like a real person.” (S18).*

Privacy and data security were further sources of concern:

*“I’m worried that the content generated by AI might be recorded by the platform or be the same as someone else’s—it does not feel safe.” (S8).*

*“I use AI to help write papers, but I do not know if the platform stores my content or might even leak it.” (S11).*

*“If someone uses AI to fake a hospitalization video, how could we tell if it’s real or not? It’s easy to be deceived.” (S19).*

#### 3.2.2 Low-quality or inaccurate outputs

Many students reported that Gen AI sometimes generated irrelevant, contradictory, or inaccurate responses:

*“Some domestic AI systems just do not understand what I’m saying. I ask a question, and the response makes no sense.” (S36).*

*“I asked it to analyze a complex case, and it told me to exercise more, then in the next sentence said I should stay in bed. Totally contradictory.” (S27).*

*“It tries to guess the answer I want and then just makes things up.” (S25).*

*“I just want the main points, but it floods me with so much information that I end up more confused.” (S24).*

Outdated content and lack of authoritative references were also frequently cited:

*“In the nursing plan it generated, there were outdated medications—that shows its knowledge base is not up to date.” (S29).*

*“Some terms it explains are completely different from what’s in our textbooks—it just confused me more.” (S8).*

*“A lot of the references it gives cannot be found. Either they do not exist or are years out of date.” (S5).*

#### 3.2.3 Technical barriers

Some students experienced language barriers, operational difficulties, and unstable systems during use:

*“Some AI systems are entirely in English—I have to use translation software, which is often inaccurate and hard to understand.” (S20).*

*“The long text it generates has a strange style and inconsistent formatting. I end up rewriting everything myself.” (S20).*

Frequent software updates, complex workflows, and access limitations increased the burden:

*“Updating an AI system means downloading several gigabytes—and I cannot use it while it updates. Then I have to relearn how to use it.” (S14).*

*“We cannot use advanced AI like ChatGPT directly in China without VPN access and paying—it’s hard to connect.” (S5).*

Some platforms had registration and setup processes that discouraged usage:

*“I once tried to use AI for a nursing care plan—it took forever just to register. Once inside, there were so many technical terms I could not figure out how to set the parameters.” (S24).*

There were also complaints about limited functionality and high usage thresholds:

*“Data sync is slow, and some functions are only available to paying members—the free version is not user-friendly.” (S19).*

*“If you do not know how to write prompts or understand code, it’s like having a fancy tool you cannot use.” (S29).*

#### 3.2.4 Cognitive and learning risks

Some interviewees noted that while Gen AI brought convenience, it may weaken independent learning, critical thinking, and communication skills:

*“It’s too fast and too comprehensive—I stop thinking for myself. After a while, I feel like I’m outsourcing my memory to it.” (S27).*

*“I’ve become a bit dependent on it. Without AI, I do not even know where to begin revising.” (S12).*

Several students admitted that using AI fostered laziness and shortcut thinking:

*“I used to organize my notes seriously, but now I just think about letting AI handle everything—I do not want to use my brain anymore.” (S35).*

Others expressed long-term concerns about its effects on human cognition and social interaction:

*“It’s like people are thinking less and less—our brains are getting lazier.” (S33)*

*“If we get used to AI-style conversation, we might lose our ability to express ourselves and communicate in real life.” (S25).*

### 3.3 Theme 3: opportunities

Participants generally believed that with continuous technological advancement and improvements in the educational environment, Gen AI holds significant potential in nursing education. Students expressed expectations for more support in terms of policies, resources, technological capabilities, interdisciplinary integration, and career development to fully realize the value of Gen AI in both learning and future professional practice.

#### 3.3.1 Policy and resource support

Many students called for a systematic and standardized guidance mechanism for AI usage to ensure compliance and sustainability in academic settings:

*“If AI had a user manual like medications, clarifying its data sources and review mechanisms, I’d feel much more confident using it.” (S11).*

For graduating students, acquiring and applying AI tools effectively was seen as an urgent need:

*“When writing my thesis, I realized how much I lacked in both technical and content knowledge. I hope there will be policy support, like skill training, to help us understand tools like ChatGPT in depth.” (S36).*

Several students emphasized the need for training in prompt engineering:

*“I heard that using the right prompts makes a huge difference, but we have never learned how. I’d appreciate prompt-related training.” (S13).*

*“Although Peking University published a tutorial on DeepSeek, it’s too general. I think there should be a guide tailored for AI use in nursing.” (S20).*

Others noted that their understanding of AI capabilities remained superficial and hoped for a broader introduction to its functions:

*“I still think of AI as just image recognition or text input. I hope there are resources to help us explore its full potential for learning.” (S31).*

Some students even proposed the creation of a professional AI platform for nursing:

*“I hope there will be an AI knowledge base specifically designed for nursing, continuously updating nursing cases, research reports, and related data—authoritative and comprehensive.” (S34).*

#### 3.3.2 Technological advancement and evolution

Most students held a positive outlook on the development of domestic AI technologies, acknowledging their rapid progress:

*“I used to envy ChatGPT abroad, but now we have our own AI tools here, and their progress is impressive.” (S32).*

Students also focused on improvements in AI’s interactive abilities:

*“AI is evolving fast—from dumb responses to now being able to analyze and generate content. It has great potential.” (S26).*

They noted that AI is increasingly embedded in everyday educational tools:

*“Phones, tablets, and many apps now have AI features—it’s already part of our daily life.” (S33).*

AI was widely regarded as a trans formative force for both education and intelligent nursing:

*“AI is a product of the big data era. Many things we could not imagine before are now possible. I think it has huge growth potential.” (S15).*

*“I believe fully functional care robots will exist in the future, providing 24/7 support to patients.” (S28).*

*“I hope that AI will eventually design personalized care plans based on individual patient needs.” (S6).*

#### 3.3.3 Interdisciplinary integration and collaboration

Many students emphasized that the integration of AI into nursing education requires close interdisciplinary collaboration:

*“I hope our school can offer more courses that combine AI with nursing. It would help us expand our thinking and learn cross-disciplinary knowledge.” (S16).*

Some recognized AI’s implications for ethics and society:

*“When using AI to make decisions, we must consider ethics—patient privacy must be protected, and the recommendations must be morally appropriate.” (S30).*

In terms of addressing regional healthcare disparities, students saw opportunities for cooperation between nursing and engineering:

*“Remote nursing can work with communications engineering to create systems so patients in remote areas can get real-time monitoring and care.” (S34).*

Others proposed integrating psychology and humanities into AI systems to enhance understanding of patients’ emotions:

*“If AI can integrate with psychology, it could help us better understand patients’ emotional states.” (S32).*

The integration of AI was also seen as a way to make course design more engaging:

*“Lectures that are just textbook-based can be boring. Adding AI could increase interaction and make learning more enjoyable.” (S10).*

#### 3.3.4 Emerging career opportunities

Students widely believed that mastering Gen AI tools would provide a significant advantage in the job market:

*“Nurses who can use AI have an edge—for example, using it for personalized health management is far more efficient.” (S30).*

Some had already observed AI’s influence on healthcare practice during internships:

*“During my ICU placement, doctors often discussed AI-assisted diagnosis. Mastering this kind of technology is a core skill of the future.” (S4).*

Others saw firsthand the rising demand for AI-competent professionals:

*“I participated in a nursing AI innovation project at school, and found many healthcare institutions looking for people who can apply AI in nursing research.” (S34).*

Students also noticed how AI may reshape the traditional nursing role:

*“I read an article saying nursing will increasingly use smart assistive tools in the future, which will improve efficiency and create new job types.” (S21).*

Additionally, students found practical help from AI in job preparation:

*“AI helped me write my resume and practice for interviews—it’s really useful for job hunting.” (S35).*

### 3.4 Theme 4: threats

Although Gen AI has demonstrated significant potential in nursing education, participants also voiced concerns about various external risks associated with its implementation. These threats primarily centered around the cost of technological adaptation, the digital divide and educational equity, the risk of job displacement, and challenges to educational quality and integrity.

#### 3.4.1 Technological adaptation and cost risk

With the rapid evolution of AI, some students expressed anxiety about their ability to keep pace with ongoing technological updates, fearing that their current knowledge would quickly become obsolete:

*“I’m worried that what we are learning now will soon be outdated and I will not be able to keep up with the technology.” (S1).*

Frequent tool upgrades and the cost of training posed additional burdens:

*“AI tools update too fast. We always have to relearn how to use new tools and sometimes even pay extra for training programs.” (S3).*

Financial pressure also limited access to high-quality AI resources:

*“Sometimes I need to pay to access research articles or plagiarism checkers—it’s too expensive for us students.” (S5).*

Some students felt alienated by the technical jargon involved in AI usage:

*“I do not understand all the terms like algorithms, models, or code. I do not have the time or energy to study that stuff.” (S18).*

Others mentioned the psychological stress brought on by constant technological demands:

*“The fast pace of AI development makes me anxious. I constantly feel like I’m falling behind.” (S26).*

#### 3.4.2 Digital divide and equity gap

The widespread adoption of Gen AI has, in some cases, widened existing disparities in access to educational resources and digital literacy. Some students voiced frustration over inequity:

*“In our school’s AI innovation project, students who come from wealthier families and know how to code built impressive models. I had neither the skills nor the resources—I could only watch.” (S21).*

Unequal tech skills within student groups affected collaboration and participation:

*“When we worked on AI-based case analysis, some group members did not even know how to enter data. It felt like we were split into two camps.” (S24).*

Regional disparities in educational resource allocation were also mentioned:

*“I heard other schools bring in experts to teach AI usage and offer hands-on training. We had to figure it all out on our own.” (S14).*

Even basic infrastructure differences became barriers to AI integration:

*“Our university does not even have an AI lab. Meanwhile, top schools use AI to simulate clinical scenarios—we have not even had a basic coding class.” (S8).*

#### 3.4.3 Job displacement risk

Students expressed varying degrees of anxiety over AI gradually replacing nursing roles. Some worried that their traditional skills might become irrelevant:

*“The terminology in AI-related papers is way beyond me. I fear that even if I study hard, I still will not be able to compete with AI.” (S31).*

Others noted that AI competence is becoming a new job market requirement:

*“Hospitals are now checking if you know how to use AI. It feels like traditional nurses are becoming less valuable.” (S20).*

Several students were unsure about their future roles amid growing AI integration:

*“I’ve seen reports from other countries using AI for basic nursing care. Are we supposed to work with AI or compete against it?” (S2).*

Some expressed fear of being directly replaced:

*“If AI can monitor vital signs, what’s the point of having nurses? We might be replaced in a few years.” (S30).*

*“Some hospitals are already using robots for nursing care. What competitiveness will undergraduate students have then?” (S29).*

#### 3.4.4 Educational integrity risk

The increasing use of AI in education has raised concerns about instructional quality and fairness. Students questioned the role of AI about traditional authority:

*“When AI and the teacher give different answers, who are we supposed to trust?” (S2).*

Academic integrity issues were also frequently mentioned:

*“Sometimes AI just copies stuff from the internet. If we are not careful, it could turn into plagiarism.” (S11).*

Students worried that over-reliance on AI could weaken their independent thinking skills:

*“If we use AI for all our assignments, no one will want to think for themselves anymore.” (S33).*

Concerns about bias in AI-generated content were also raised:

*“Gen AI might amplify the bias in its training data, which could affect fairness in education.” (S31).*

Some students were unsure about ownership of AI-generated content:

*“If AI writes something, who owns it? Could it lead to copyright problems?” (S5).*

Lastly, the blurred line between AI-generated and student-written work was a source of confusion:

*“It’s hard to tell the difference between AI-generated and original work. That might affect how our actual abilities are assessed.” (S36).*

## 4 Discussion

**Figure 4:**
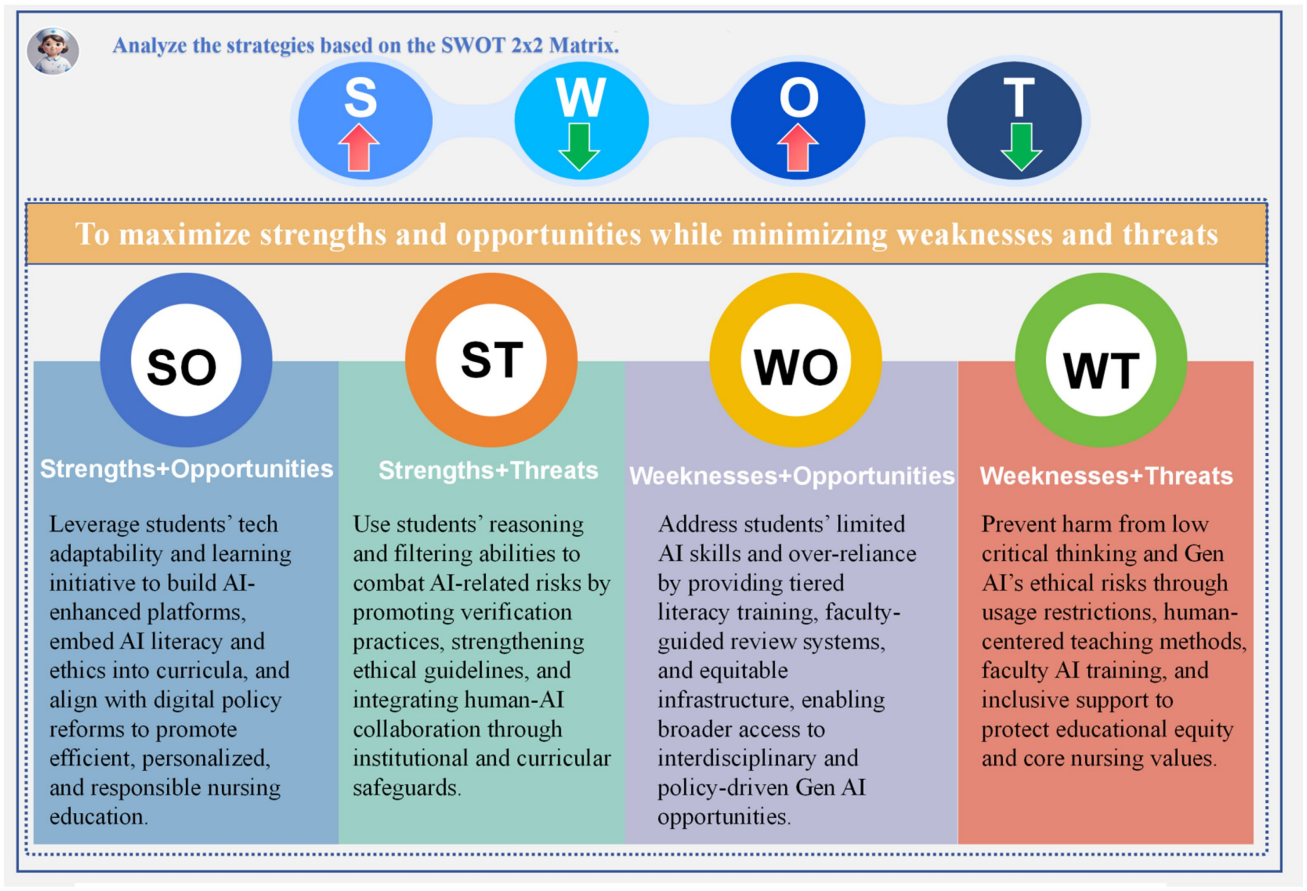
Analyze the strategies based on the SWOT 2×2 matrix.

This study found that undergraduate nursing students widely use Gen AI tools, primarily for academic purposes such as problem-solving, course support, and academic writing, which is consistent with the findings of Pham TD (3). In addition, participants showed a clear preference for domestic platforms. Most students reported frequent daily use, typically for 15–30 min per session, indicating a strong reliance on these tools for learning. Through in-depth interviews, the study systematically identified the strengths, weaknesses, opportunities, and threats (SWOT) associated with Gen AI use. Overall, students expressed high recognition of Gen AI’s value, particularly in personalized learning support, skills training, and enhancing learning efficiency. However, challenges such as ethical and legal risks, information inaccuracy, technical barriers, and cognitive dependence were also noted. Externally, policy support, technological advancement, interdisciplinary collaboration, and career development presented key opportunities, while adaptation pressure, educational inequities, job displacement risks, and concerns over teaching quality emerged as potential threats. While students showed strong acceptance of Gen AI, they also remained cautious about associated risks. Based on the SWOT analysis framework, the study constructed a 2×2 strategic matrix that clearly defines SO, ST, WO, and WT strategies. It emphasizes how to maximize the use of strengths and opportunities while minimizing weaknesses and threats to develop effective strategies (Figure 4). This matrix not only offers theoretical insights but also provides practical pathways for the ethical and effective integration of Generative Artificial Intelligence (Gen AI) into nursing education.

### 4.1 Leveraging strengths to seize opportunities (SO strategy)

Undergraduate nursing students demonstrate strong learning abilities, information integration skills, and technological adaptability when using Gen AI. Research shows that students’ technology acceptance and learning initiative are key factors driving the effectiveness of AI education. These internal strengths should be fully utilized, combined with educational digital transformation and AI development strategies, to create a favorable external environment for integrating Gen AI into nursing education.

In terms of instructional design, it is recommended to develop AI teaching platforms that integrate intelligent recommendations, virtual case simulations, and real-time feedback. Studies have confirmed that such platforms can effectively improve learning efficiency and clinical practice skills (28). For example, Yang et al. highlighted that AI-driven situational simulations help enhance students’ judgment and operational abilities (29).

Curriculum development should systematically incorporate AI literacy education, including prompt engineering, generative writing, and clinical decision-making simulations, to enhance students’ technical application skills and professional judgment. Related studies also support integrating AI into curricula to cultivate nursing professionals with interdisciplinary competencies (30). Additionally, strengthening education on AI ethics, data security, and academic integrity can improve students’ sense of responsibility and critical thinking (31–33).

At the policy level, it is necessary to formulate AI teaching guidelines specifically for nursing programs, promote the digital upgrade of educational resources, and especially enhance technical support and platform access in under-resourced areas. Research indicates that balanced allocation of educational resources is a fundamental guarantee for the widespread adoption of AI education (34). Furthermore, Shishehgar et al.’s systematic review emphasizes that embedding AI training in health curricula not only facilitates curriculum reform but also requires attention to ethical standards and cost-effectiveness, further supporting the integrated curriculum and policy strategies proposed in this study (21, 32).

### 4.2 Leveraging strengths to mitigate threats (ST strategy)

While Gen AI significantly enhances learning efficiency and access to resources, its unstable content generation, increasing ethical concerns, and the widening digital divide continue to pose potential threats to nursing education (35, 36). Students’ critical thinking skills and digital adaptability can serve as key assets in addressing these challenges (37).

In educational practice, students should be guided to verify AI-generated content through cross-platform comparison and evidence-based validation, thereby improving their abilities in information filtering, logical reasoning, and risk perception. In particular, courses such as nursing ethics and research methodology can incorporate case-based analytical tasks to promote the integration of professional judgment with technological application (38, 39).

At the institutional level, clear guidelines on the ethical use of AI should be established, with explicit restrictions on its use in core knowledge construction and competency assessment to prevent over-reliance and blurred accountability (37). Educators should use case analysis and classroom discussions to reinforce students’ understanding of AI as a tool, fostering rational use and collaborative human-AI interaction.

Existing studies have noted that Gen AI may produce fabricated information, algorithmic bias, or factual inaccuracies. Without an effective human review mechanism, such issues can compromise content quality and academic integrity (35, 40). Therefore, it is necessary to promote coordinated efforts in policy development, curriculum integration, and pedagogical guidance (41). By leveraging students’ cognitive and technological strengths, a multidimensional risk management framework can be established to support the responsible integration of AI into nursing education (37, 38, 42).

### 4.3 Overcoming weaknesses to exploit opportunities (WO strategy)

Nursing students often face internal challenges in using Gen AI, such as limited operational proficiency, difficulty in verifying information, and a tendency toward over-reliance (12, 25). However, with supportive educational policies, improved technological infrastructure, and growing interdisciplinary integration, these weaknesses are increasingly addressable (40, 43).

It is recommended to implement tiered AI literacy courses at the undergraduate level, covering prompt design, interaction optimization, content validation, and ethical reasoning, to enhance students’ technical competence and risk awareness (44). At the same time, a faculty-led content review mechanism should be established to ensure the professional accuracy of AI-generated materials used in teaching.

To promote equitable access, educational authorities should develop AI platforms tailored to nursing education, integrating standardized manuals, case libraries, and review workflows, with particular support for resource-limited regions (45). Studies indicate that AI literacy significantly improves students’ sense of learning control, while faculty involvement is essential for maintaining educational quality (46, 47). At a broader level, equitable resource allocation and regional collaboration mechanisms are fundamental to the sustainable and inclusive development of AI in education.

### 4.4 Overcoming weaknesses to avoid threats (WT strategy)

Students’ limited critical thinking skills, combined with ethical risks and unstable content quality from Gen AI, may adversely affect nursing education (42). To mitigate these threats, a tiered course management system should classify AI tool use into prohibited, restricted, and open levels based on teaching stage and course nature, preventing over-dependence during core competency development. Simultaneously, non-AI teaching activities such as role-playing, scenario simulations, and communication training should be strengthened to ensure clinical and humanistic competence.

Faculty development requires establishing specialized training programs to enhance teachers’ AI instructional capabilities, ensuring controlled and effective technology integration (48). Furthermore, attention must be given to access issues faced by disadvantaged students by providing shared devices and platforms, fostering an inclusive and equitable learning environment that alleviates digital divide-induced disparities and anxieties.

To ensure that the humanistic qualities of nursing education, centered on empathy and judgment, are not replaced, it is essential to strengthen support for technologically disadvantaged groups (44). Through evidence-based guidelines developed from multidisciplinary consensus, universities can effectively regulate the use of AI technologies, overcome weaknesses and threats, and promote the safe and rational application of Gen AI in nursing education (45, 49, 50).

Moreover, when applying AI tools in clinical nursing education, teachers should adopt a “wise use” approach to promote students’ clinical competencies (51). AI-based simulations, decision-support systems, and virtual patients can facilitate the development of clinical judgment, communication, and reflective skills (52). However, educators need to guide students to critically interpret AI suggestions rather than relying on them passively, ensure patient safety is prioritized, and incorporate ethical and humanistic values into AI-assisted learning activities (37, 53).

## 5 Conclusion

This study innovatively applied the SWOT analytical framework, integrating prior quantitative findings with in-depth qualitative interviews to systematically examine the experiences and perceptions of undergraduate nursing students in western China regarding the use of Gen AI. The results revealed several perceived strengths, including personalized learning support, enhanced study efficiency, assistance with academic writing, and the development of clinical reasoning skills.

At the same time, the study identified key challenges, such as high technical barriers, increased dependency on AI, ethical concerns, and doubts about the accuracy and reliability of AI-generated content. While most students held positive attitudes toward Gen AI, recognizing its role in enhancing learning motivation and knowledge acquisition, they also expressed concerns about its long-term impact, particularly in areas such as cognitive development, academic integrity, and professional roles.

To inform educational practice and policy, the study developed a 2 × 2 strategic matrix based on SWOT findings, proposing four strategic pathways: (leveraging strengths to seize opportunities), ST strategies (leveraging strengths to mitigate threats), WO strategies (overcoming weaknesses to exploit opportunities), and WT strategies (overcoming weaknesses to avoid threats). This structured framework offers guidance for the integration of Gen AI into nursing education, enabling stakeholders to maximize its potential while addressing associated risks and challenges effectively.

## 6 Limitations

This study was conducted at a single medical university in western China. Although participants came from diverse backgrounds, the limited geographic scope may affect the generalizability of the findings. The sample size was relatively small (*n* = 36), and students with limited exposure to AI may be underrepresented. Data collection relied on self-reported narratives, which may introduce individual bias. Moreover, the study did not include key stakeholders such as faculty members, clinical instructors, or administrators, limiting a comprehensive understanding of AI integration in education. Future research should broaden the sample scope and incorporate multiple perspectives to enhance external validity and practical relevance.

## Data Availability

The original contributions presented in the study are included in the article/supplementary material, further inquiries can be directed to the corresponding author.
